# Diurnal variation in the human skin microbiome affects accuracy of forensic microbiome matching

**DOI:** 10.1186/s40168-021-01082-1

**Published:** 2021-06-05

**Authors:** David Wilkins, Xinzhao Tong, Marcus H. Y. Leung, Christopher E. Mason, Patrick K. H. Lee

**Affiliations:** 1 School of Energy and Environment, City University of Hong Kong, Hong Kong SAR, China; 2 Department of Physiology and Biophysics, Weill Cornell Medicine, New York, NY USA; 3 The HRH Prince Alwaleed Bin Talal Bin Abdulaziz Alsaud Institute for Computational Biomedicine, Weill Cornell Medicine, New York, NY USA; 4 The WorldQuant Initiative for Quantitative Prediction, Weill Cornell Medicine, New York, NY USA; 5 The Feil Family Brain and Mind Research Institute, Weill Cornell Medicine, New York, NY USA

**Keywords:** Built environment, Forensics, Microbiota, Skin, Surface

## Abstract

**Background:**

The human skin microbiome has been recently investigated as a potential forensic tool, as people leave traces of their potentially unique microbiomes on objects and surfaces with which they interact. In this metagenomic study of four people in Hong Kong, their homes, and public surfaces in their neighbourhoods, we investigated the stability and identifiability of these microbiota traces on a timescale of hours to days.

**Results:**

Using a Canberra distance-based method of comparing skin and surface microbiomes, we found that a person could be accurately matched to their household in 84% of tests and to their neighbourhood in 50% of tests, and that matching accuracy did not decay for household surfaces over the 10-day study period, although it did for public surfaces. The time of day at which a skin or surface sample was taken affected matching accuracy, and 160 species across all sites were found to have a significant variation in abundance between morning and evening samples. We hypothesised that daily routines drive a rhythm of daytime dispersal from the pooled public surface microbiome followed by normalisation of a person’s microbiome by contact with their household microbial reservoir, and Dynamic Bayesian Networks (DBNs) supported dispersal from public surfaces to skin as the major dispersal route among all sites studied.

**Conclusions:**

These results suggest that in addition to considering the decay of microbiota traces with time, diurnal patterns in microbiome exposure that contribute to the human skin microbiome assemblage must also be considered in developing this as a potential forensic method.

**Video Abstract**

**Supplementary Information:**

The online version contains supplementary material available at 10.1186/s40168-021-01082-1.

## Introduction

A central aim of forensic science is to accurately identify people from the trace evidence they leave behind whilst moving through and interacting with an environment. Because people carry skin microbiomes which can be individually distinctive [1] and to some degree stable across time [2], and which can be transferred to surfaces through direct contact, the comparison of skin and surface microbiota (hereafter ‘microbiota matching’) has been recently investigated as a potential forensic identification method [3–5] and compared to the long-established forensic technology of fingerprinting [6, 7]. In research contexts, this method has been successfully used to match people to objects they have touched including computer keyboards and mice [7], mobile phones [8, 9] and artificial crime scenes [10], to match recently deceased people to objects found at the scene of death [6] and to match people to the households in which they live [11–13].

Despite these successful demonstrations, the analogy between microbiota traces and fingerprints is misleading. Unlike fingerprints, which are generally stable for a person’s entire life and which can persist on a surface unchanged for months to years, a person’s skin microbiome can shift over weeks or months [14, 15] due to factors including the person’s physiology, their environment, dispersal from external sources, and stochastic assembly processes (drift), although generally these changes over time are smaller than intrapersonal differences [16]. With the exception of physiology, traces deposited on inert surfaces are susceptible to the same forces, and may be exposed to ongoing microbial deposition from other sources. These forces may occlude or eliminate a forensic microbiota trace. In a previous study of microbiota matching of people to their homes over a time scale of months, we found that taxa that were more useful in identifying people were more likely to be lost from both skin microbiomes and surface traces over time, and less likely to be dispersed from skin to surfaces in the first place [11]. Minor, low-abundance taxa may not only be more valuable in identifying individuals compared to abundant taxa [11, 17], but may also be more susceptible to elimination and more likely to be the result of deposition events (‘transients’) rather than being well adapted to the skin environment and established in microbial community networks (‘residents’). The nature of these low-abundance taxa, the processes that cause the loss of identifying features and the time scale on which this loss occurs are poorly understood. In this study, we performed a metagenomic survey of samples taken from the palms of four people in Hong Kong, household surfaces within their homes, and hand rails in public spaces near their homes each morning and evening for ten consecutive days, to investigate the time scale on which this loss of identifying microbial features occurs and to characterise the community and species properties that contribute to identifiability and to trace deterioration.

## Results and discussion

### Overview of samples and taxonomic composition

**Fig. 1:**
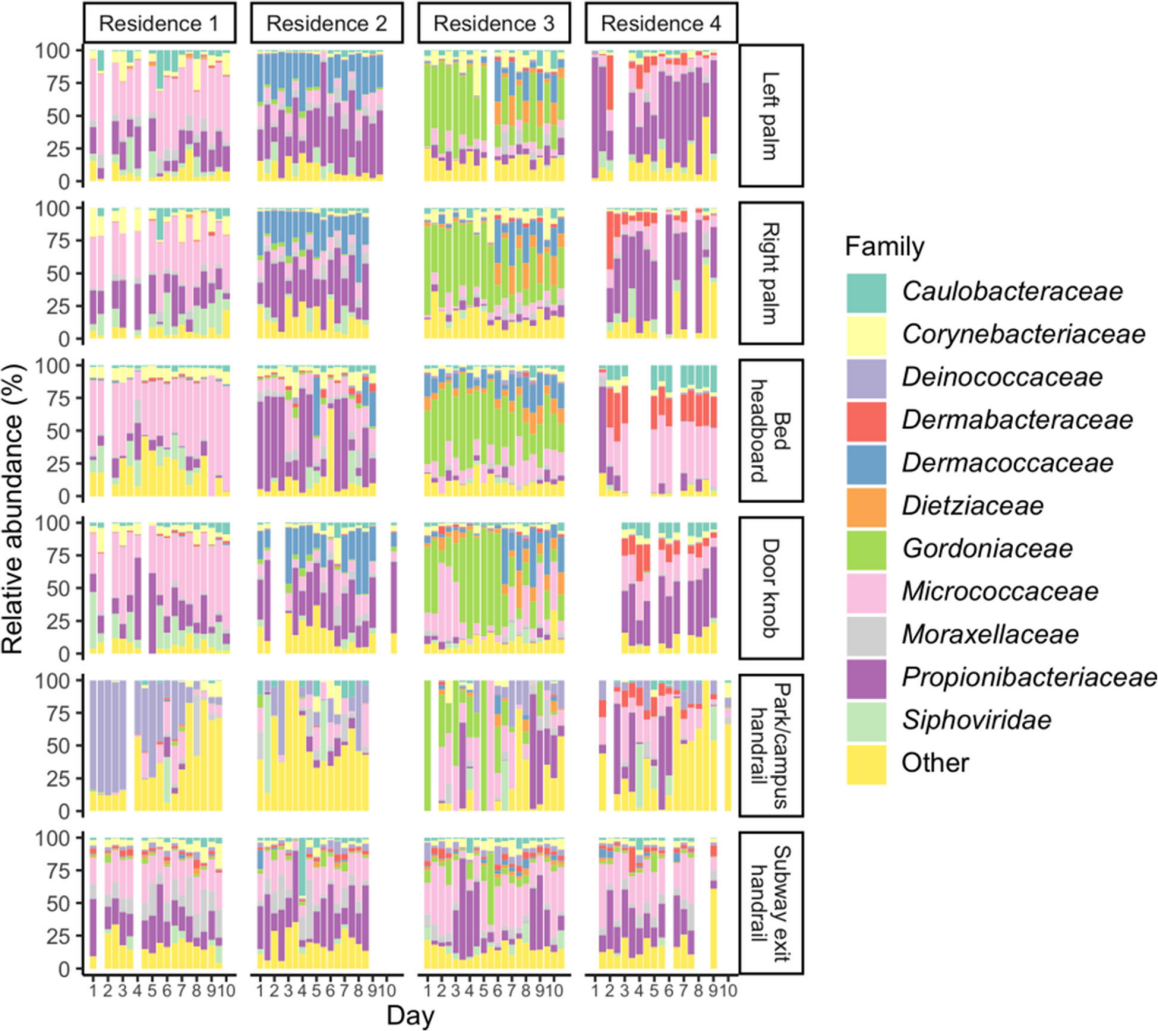
Relative abundances of microbial families on skin, household surface and public surface sites across the study period. Each day comprises a morning (left bar) and evening (right bar) sample. Some samples were excluded from the study due to sequencing failure (see Table S1)

We collected morning and evening microbiota samples across a 10-day sampling period from the palms of four people in Hong Kong, from the bed headboards and internal front door knobs in their homes, and from handrails in a subway entrance and a park or campus near their homes. The study participants all lived alone and in different regions of Hong Kong. We surveyed the microbial communities present in each sample with metagenomic sequencing. Although this metagenomic approach captured members of the Archaea, Eukarya and some viruses, Bacteria were the most abundant domain across all samples of all types (skin: mean abundance 93%, SD 6.9%; household surface: mean 95%, SD 8.8%; public surface: mean 95%, SD 11%). The pooled microbial abundance across all skin samples was dominated by a shared set of known skin commensals, most notably the families *Propionibacteriaceae*, *Micrococcaceae* and *Moraxellaceae* (Fig. 1). However, each person’s skin microbiome had at least one notable bacterial family that was present at higher abundance on that person than on other people, creating individual taxonomic profiles that were similar between a person’s left and right palm and generally maintained across the 10 days of sampling. Person 1 was noted to have a high abundance of *Micrococcaceae* compared to the other people in the study; person 2 was characterised by *Dermacoccaceae*; person 3 a mix of families including *Gordoniaceae*, *Dietziaceae* and *Dermacoccaceae*; and person 4 *Dermabacteraceae*. These distinctive profiles were reflected in the surface microbiomes from the respective homes, with residence 1 having a substantial abundance of *Micrococcaceae*, residence 2 a high abundance of *Dermacoccaceae* on the door knob, residence 3 a high abundance of *Gordoniaceae* on both the door knob and bed headboard and residence 4 a high abundance of *Dermabacteraceae*. This pattern of locality-specific families was not as evident for the public surface samples, which are likely to reflect microbiota input pooled from the local population and environmental sources.

The overall abundance of *Propionibacteriaceae* in skin samples (mean relative abundance 27%, SD 23% across all skin samples) was higher than in our previous 16S rRNA gene-based survey of a similar group of Hong Kong households and occupants [11]. This is likely because the 515F/806R primer pair used in the previous study is biassed against the phylum *Actinobacteria* [18] of which the family *Propionibacteriaceae* is a member, and highlights a benefit of the metagenomic approach in capturing a more representative sample of the species present.

### Human skin as the major microbial source for surface communities

**Fig. 2:**
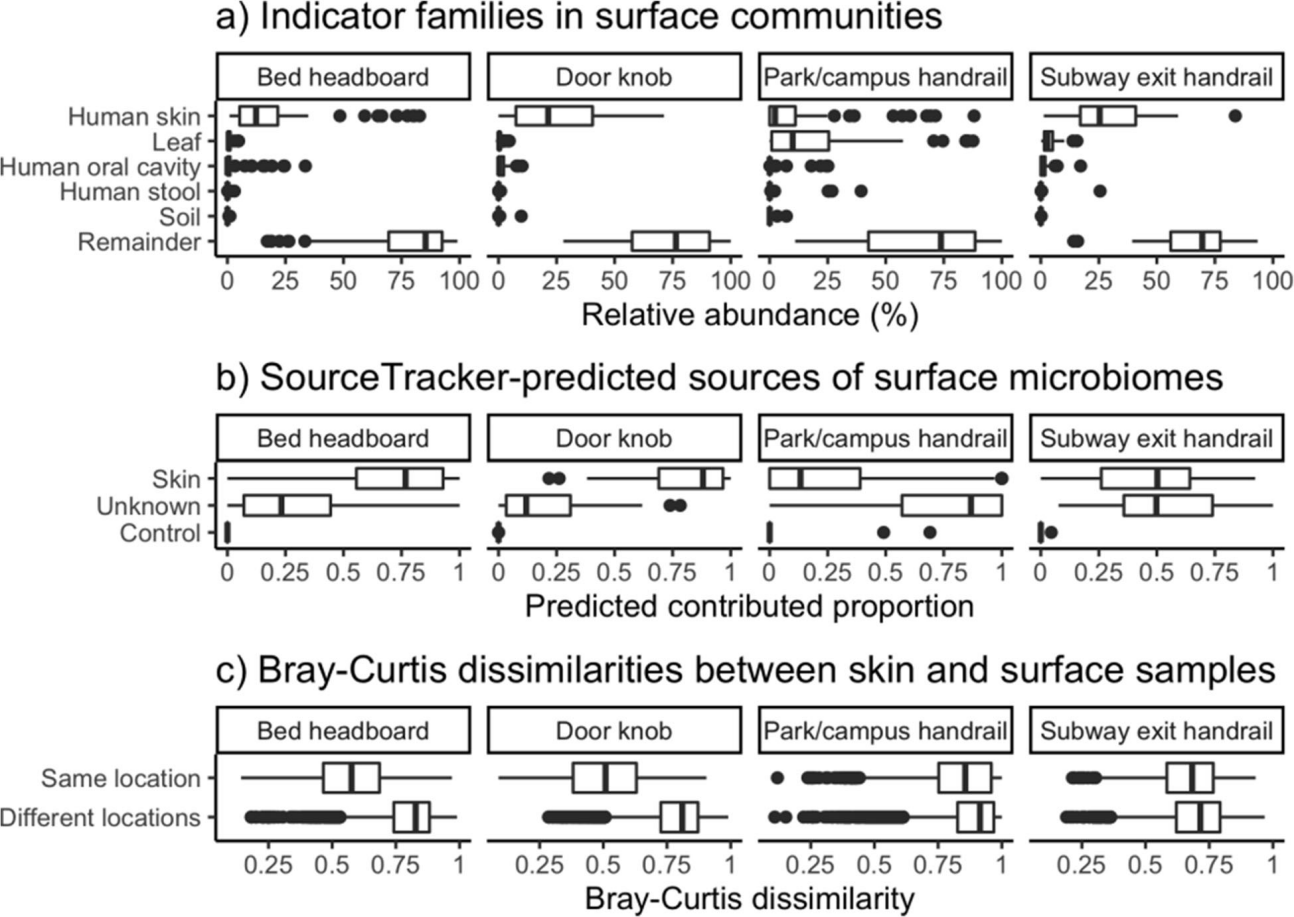
(**a**) Abundances of bacterial families identified by Dunn et al. [19] as indicators of human and environmental sources. (**b**) SourceTracker predictions for microbiota sources. (**c**) Bray-Curtis dissimilarities between skin and surface communities

A key premise of microbiota matching as a forensic method is that there is significant transfer of microbiota from a person’s skin to surfaces they touch. We used three methods to confirm that human skin was the major source of microbiota on the sampled household and public surfaces. First, we examined the relative abundances of the families identified by Dunn et al. [19] as indicators of human (skin, oral, stool) vs. environmental (leaf, soil) sources. With the exception of ten of the public surface samples, in which ‘leaf’ was the major microbial source identified and ‘human skin’ the second-major source, the highest-abundance identified source in every surface sample was human skin (Fig. 2a). Second, we used SourceTracker [20] to identify whether human skin or negative control samples were the most likely sources for surface communities (Fig. 2b). Again with the exception of public surfaces, human skin was predicted to be the major source for samples of all types, although the predicted proportion of skin as a source for household surface samples (mean 75%, SD 25%) was higher than for public surface samples (mean 36%, SD 31%), with a much higher proportion of ‘unknown’ sources in these environments. As the ‘skin’ source is determined only from the skin of study participants, this ‘unknown’ source potentially includes skin inputs from other people who have touched these public surfaces. Third, we compared the Bray-Curtis dissimilarities between pairs of skin and surface samples taken from the same location with pairs from different locations (Fig. 2c). Communities at all surface sites were significantly more similar (Mann-Whitney p < 0.05) to skin communities from the same location than communities from different locations, although the magnitude of the difference was larger for household surfaces than for public surfaces. Together, these results confirm the now well-established finding that the microbiomes on frequently touched surfaces both in homes and in public spaces are assembled substantially from deposition of microbiota from human skin, and in particular resemble the skin of the household occupants or local residents.

### Performance of microbiota matching methods

**Fig. 3:**
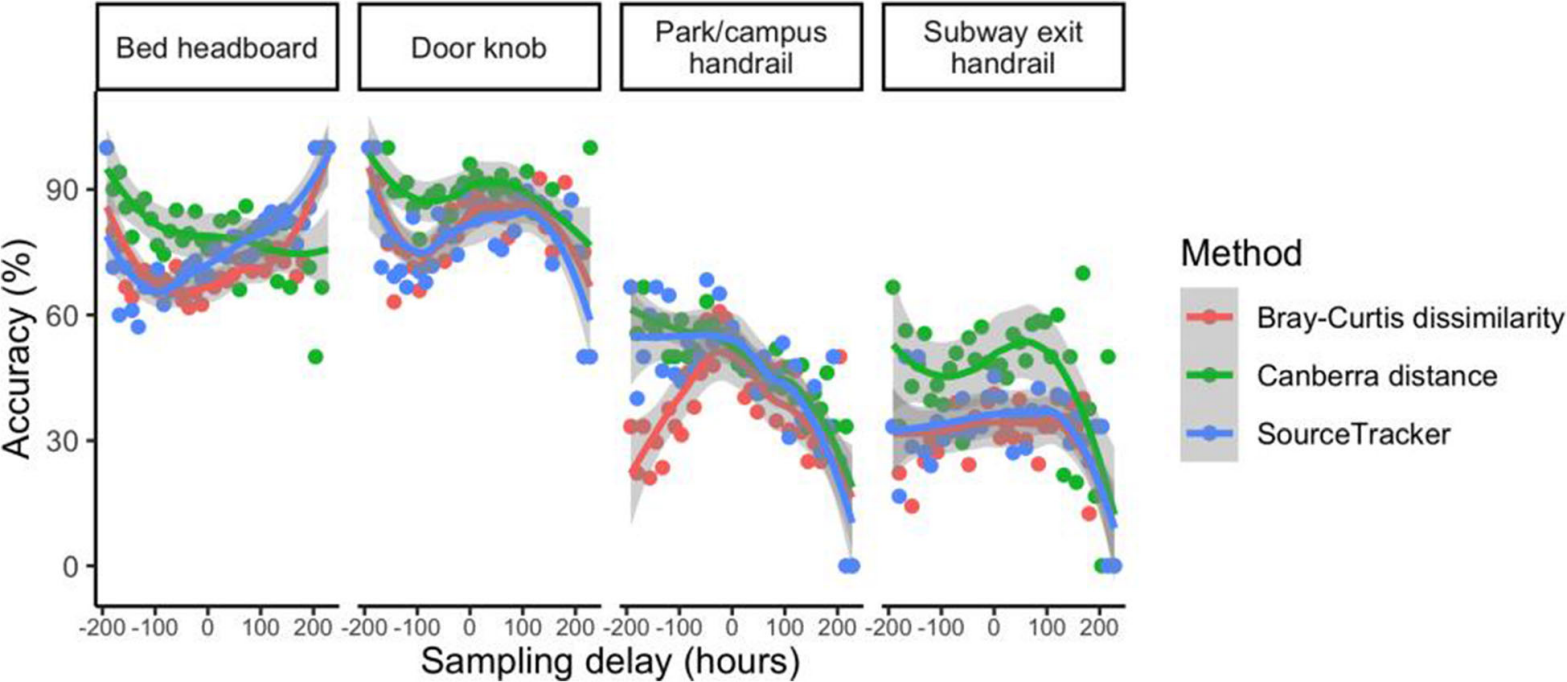
Microbiota matching accuracy with the SourceTracker, Canberra distance and Bray-Curtis dissimilarity methods. Points represent the percentage of matching attempts for surface query samples at each site and time point that were matched to the correct location. Solid lines and shaded areas represent the LOESS (locally estimated scatterplot smoothing) moving average and 95% confidence interval respectively. Sampling delay represents the approximate time in hours between the collection of the reference samples and query sample; a negative value indicates that the query sample was collected before the reference samples

Microbiota matching aims to accurately identify the person who has touched a surface by comparing the surface’s microbiome (hereafter the ‘query’) to the skin microbiomes of a group of people (hereafter the ‘pool’ of ‘references’). Previous studies have used a range of methods to achieve this, including SourceTracker prediction of the sources of surface microbiota [11, 13], shared taxonomic composition between query and references [9] and community dissimilarity between query and references [7, 17]. We compared matching with SourceTracker, which we have used previously to match a similar Hong Kong cohort to their household surfaces with 67% accuracy [11], to matching by minimum Canberra distance or Bray-Curtis dissimilarity [17] (Fig. 3). Included in this comparison were query and reference samples taken at the same time as well as samples taken at different times (‘sampling delay’). Overall, the Canberra distance method produced the most accurate matches, with 84% accuracy for household surface and 50% for public surface samples across all matching attempts, significantly higher (χ squared p < 0.05) than the SourceTracker method (77% accurate for household surfaces, 42% for public) and the Bray-Curtis dissimilarity method (75% household, 38% public). Of the 172 combinations of query site and sampling delay for which each matching method was tested, the Canberra distance method had the highest or equal-highest matching accuracy in 102 combinations. This is consistent with the report of Watanabe et al. [17] that the Canberra distance outperforms other distance and dissimilarity measures; as noted by Meadow et al. [21], this is likely because the Canberra distance is more influenced by low-abundance taxa that drive the individuality of grossly similar microbial habitats such as skin or surfaces. We used the Canberra distance method for all further matching analyses.

### Temporal effects on matching accuracy

We examined the effect of sampling delay, in which a surface ‘query’ sample is taken some time before or after a skin ‘reference’ sample, on the accuracy of microbiota matching. As our study took place over a 10-day period with morning and evening samples taken on each day, the range of sampling delays examined was between zero (samples taken at the same time) and 10 days (query sample taken 10 days after reference sample, or vice-versa), with increments of approximately 12 h. For household surface queries, there was no significant relationship between absolute sampling delay and matching accuracy (Fig. 3; Spearman’s ρ, p > 0.05), in contrast with our previous study where matching accuracy declined significantly for household surface queries collected several months before or after the reference pool [11]. This suggests that household surface traces up to at least 10 days old have not yet been substantially affected by the processes that degrade the microbial matching utility of older traces. By contrast, for public surface queries, there was a strong (Spearman’s ρ = –0.73 for public handrail, –0.51 for subway exit handrail) and significant (p < 0.05) negative relationship between absolute sampling delay and matching accuracy (Fig. 3).

There was also a substantial and significant relationship between the time of day at which the query sample and/or pool of reference samples was taken and matching accuracy (Fig. S1). For household surfaces, queries collected in the evening had a significantly higher accuracy rate than morning queries (χ squared p < 0.05), whilst skin reference pools collected in the morning had a significantly higher accuracy rate than those collected in the evening (p < 0.05). For public surface sites, however, morning queries had a significantly higher accuracy rate than evening queries (p < 0.05), whilst there was no significant difference in accuracy rate between morning and evening references. This suggests that there are significant differences in the temporal dynamics underlying the similarity of people’s skin and home microbiota environments compared to skin and public surfaces, with divergence between skin and public surface microbiomes occurring significantly faster than between skin and household surface. Further, it suggests that these dynamics, which include processes of microbial dispersal between sites as well as the elimination of dispersal traces, are not uniform throughout the day but are in some way influenced by the diurnal cycle.

### Diurnal variation in skin and surface microbiomes

**Fig. 4:**
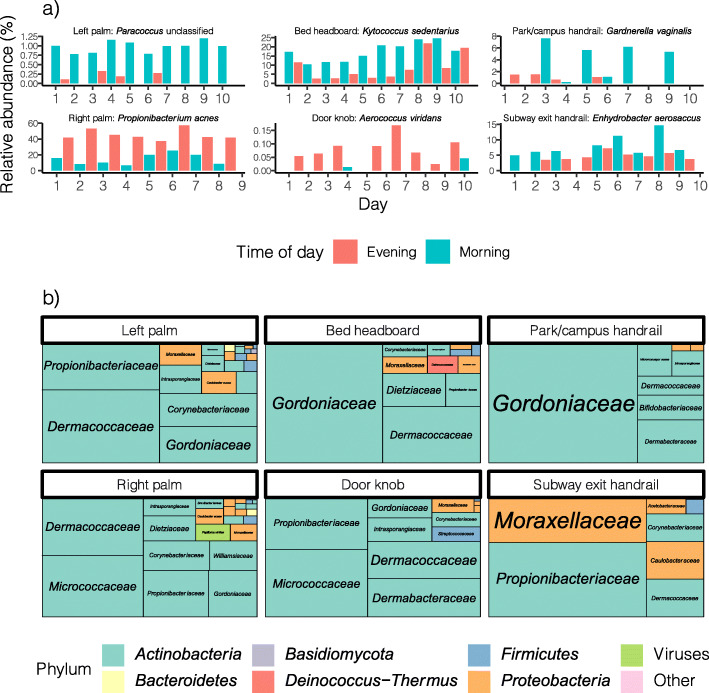
(**a**) Abundances of selected diurnally varying species over the study period. The species with the highest seasonality strength at each site is shown as an exemplar of the diurnal pattern. (**b**) Treemap showing taxonomic composition of significantly diurnal species at each site. Each tile represents a taxonomic family, with the area of the tile proportional to its mean relative abundance at that site across all locations. The fill colour of each tile represents its phylum

We noted that some taxa at both skin and surface sites appeared to have a diurnal abundance pattern, with relative abundance consistently higher in either the morning or evening. Time of day was a significant contributor to variance at the community level, with a significant difference between morning and evening communities across all species and sites (PERMANOVA p = 0.008). To better understand this diurnal variation and how it may help account for the observed time-of-day effects on microbiome matching, we investigated the identity and abundance patterns of these diurnally varying taxa. The abundance time series for each species at each site and location was decomposed into trend, random and seasonal components, with the seasonal component having a period of 1 day (i.e. a single diurnal cycle). The strength of the seasonal component (*F*_*S*_) was defined as its variance relative to the variance in the random component, and the significance of this strength assessed with 999 random permutations of the species abundances. With this method, we identified a total of 160 combinations of species, site and location where the species exhibited a significant (p < 0.05) pattern of diurnal variation (Fig. 4). The proportion of species identified as significantly diurnal, as well as the *F*_*S*_ of those species, was highest at skin sites (14% of all combinations of species, site and location, mean *F*_*S*_ = 0.40), followed by smaller proportions at household surfaces (6.0%, mean *F*_*S*_ = 0.33) and smaller again at public surfaces (2.1%, mean *F*_*S*_ = 0.29). Among significantly diurnal species, the difference in relative abundance between morning and evening was on average largest at skin sites (mean difference in relative abundance 3.3%, SD 6.4%), followed by public surfaces (mean 3.0%, SD 9.4%) and household surfaces (mean 2.5%, SD 5.7%).

Before examining biological explanations for these diurnal patterns, we first considered whether they could be artefactual or due to error. One possible source of an artefactual diurnal pattern would be a systematic difference in the processing of morning and evening samples, such as the introduction of a contaminant species though mishandling, or a bias for or against some species due to differences in DNA extraction technique. Because such a systematic difference would be expected to apply similarly to samples from the four locations, which were processed in parallel in the same laboratory and using the same equipment and reagents, we examined the number of significantly diurnal species at each site which were found in only one location. For all sites, the majority of significantly diurnal species were unique to a single location (72% of species at left palm, 75% at right palm, 100% at bed headboard, 64% at door knob, 80% at public handrail, 100% at subway exit handrail), a pattern not consistent with a systematic methodological error. We also examined the proportion of species with a significantly diurnal pattern that were identified in the negative control samples. The majority (75%) of significantly diurnal species across all samples were absent from all the negative control samples, although this proportion was smaller than that of non-diurnal species of which 94% were absent from the negative controls.

We also examined the possibility that this effect may be driven by input into our metagenomes of genetic material from reagent contamination, in spite of the decontamination steps taken (see the “Methods” section). In particular, if there was a systematic difference in the total biomass in morning compared to evening samples at the DNA extraction and sequencing stages, reagent contamination would proportionally contribute a greater proportion of the observed species abundance in the lower-biomass samples. We note that many of the genera found to exhibit a significant diurnal pattern, such as *Janibacter*, *Microoccus* and *Dietzia*, have previously been reported as common DNA extraction reagent contaminants [22], although these genera are also plausible components of human skin and environmental microbiomes. We re-ran the time series decompositions for all samples with all species that appeared in our negative control samples removed. Of all the species that had been initially identified as significantly diurnal, 25% (n = 40) were removed because they were present in the negative controls; of the remainder, 17.5% (n = 28) were no longer found to be significantly diurnal, perhaps due to compositional effects (see next paragraph ), whilst 57.5% (n = 92) remained significantly diurnal. Considering that many of the species identified in the negative controls were likely to be genuinely present in the sampled sites, these results do not support a major contribution of reagent contamination to the observed patterns of diurnal variation.

Another possible cause for an artefactual diurnal pattern is the compositional effect of normalising species counts in each sample to produce relative abundances [23]. Relative species abundances within a sample are not independent: an increase in the relative abundance of one species proportionally reduces the relative abundances of all others, even if the absolute abundances of the other species remained unchanged. Thus, it is possible that even a single high-abundance species that has a genuine diurnal pattern could cause other species within the same sample to falsely appear to have complementary diurnal patterns. We examined the number of significantly diurnal species at each site that had their abundance peak in the morning vs. evening, across the 24 combinations of site and location. Of these 24 combinations, five had no diurnal species, seven had diurnal species that all exhibited their highest abundance at the same time of day (i.e. all during the morning or during the evening), nine had a mix of diurnal species most abundant in the morning and in the evening, and the remaining three had a single species most abundant at one time with the remainder most abundant at the other time. The pattern in these latter three sites is potentially consistent with a compositional effect, with a true diurnal pattern in a single species driving an apparent pattern in the others. To examine this possibility further, the species with the singular diurnal pattern was removed from these three sites, species relative abundances were re-normalised, and the time series decompositions were re-run to determine the effect on the other diurnal species. At one of the sites, there was no difference in the number of diurnal species, one site had one (of eight, excluding the intentionally removed species) fewer significantly diurnal species after the re-analysis, and the last site had no significantly diurnal species (from three previously). Overall, whilst the possibility of a compositional effect at two of these three sites could not be excluded, there was no evidence of a compositional effect driving the diurnal pattern for the majority of sites.

To better characterise the species that exhibit a diurnal pattern, we first examined the relationship between diurnality and abundance. Diurnal species (mean relative abundance 3.1%, SD 6.5%) were significantly (Mann-Whitney p < 0.05) more abundant than non-diurnal samples (mean 0.72%, SD 2.9%), excluding samples in which a given species was absent. Abundance was also positively correlated with the *F*_*S*_ of diurnal species (Spearman’s ρ = 0.39, p < 0.05). It is notable that this positive relationship between seasonality and abundance exists in spite of the fact that diurnally varying species necessarily have significantly reduced abundance in around half of the samples in a given time series. We next examined the taxonomic composition of the diurnal species. By relative abundance, diurnal species at all sites were overwhelmingly dominated by the phylum *Actinobacteria*, and particularly the families *Propionibacteriaceae*, *Micrococcaceae*, *Gordoniaceae* and *Dermacoccaceae* (Fig. 4b). These families were noted to correspond well to the families that characterised the skin and surface samples for a particular location; for example, the family *Gordoniaceae* was unusually abundant in skin, household surface and public surface samples from location 3 (Fig. 1).

Whilst this study was not designed to assess physiological or environmental factors that may contribute to this diurnal pattern, we evaluated whether microbial dispersal was a potential or partial explanation. We hypothesised that microbial dispersal from public surfaces to hands occurs primarily during the daylight hours, when people are likely to be out of their homes and in public spaces, and is followed by a ‘normalisation’ of the skin microbiota overnight driven by bathing, handwashing and interaction with household objects and surfaces that act as reservoirs of the household occupant’s normal skin microbiome. This hypothesis presumes that the major route of dispersal among the three types of sites is from public surfaces, which represent pooled human skin microbiota as well as environmental inputs, onto skin whilst people are out in public spaces, and subsequently onto surfaces within people’s private homes. This is consistent with the observed proportions of diurnal varying species at sites of each type, with the fixed public pool having the fewest diurnal species with the weakest *F*_*S*_, skin having the most diurnal species with the strongest *F*_*S*_, and household sites falling in between. This hypothesis would also explain why morning skin references had significantly higher accuracy rates than evening references when matching against household queries, as the evening references were taken at the end of a day of dispersal from public surfaces, whilst morning references were taken at the end of the period of normalisation. It may also explain why morning public surface queries had marginally but significantly higher accuracy rates than evening queries, as these queries were taken during the daytime hours when people were likely out and interacting with public surfaces. However, it does not explain why household surface evening queries had higher accuracy rates than morning queries (Fig. S1).

To test this hypothesis, we generated Dynamic Bayesian Networks (DBNs) for all taxonomic families present at each location, modelling the effect of the abundance of each family at a given site on the abundance at other sites after one time point increment (i.e. morning to evening, or evening to the following morning, approximately 12 h). In these networks, a node represents a site at a particular location (e.g. ‘subway exit handrail at location 2’ or ‘left palm at location 4’), whilst an edge from one node to another represents a causal relationship between the abundance of a family at the first site (the ‘parent’ node) and the abundance of the same family at the second site (the ‘child’ node) after one time point increment, implying microbial dispersal from one site to another [13]. Forty-seven percent of the DBNs were generated with at least one edge. Public surface nodes were significantly (Kruskal-Wallis p < 0.05) more likely to be parents than nodes of other types, yielding 599 edges in total across all networks, compared to 525 edges from skin parent nodes and 476 from household surface parent nodes. Skin nodes were significantly (Kruskal-Wallis p < 0.05) more likely to be child nodes than other types, with 597 edges ending at skin nodes, compared to 522 ending at household surface nodes and 481 at public surface nodes. The single most common edge type was from a public surface node to a skin node, comprising 261 edges in total. This strongly supports dispersal from public surfaces to skin at the approximately 12-h time scale as the major dispersal route between the three types of sites studied. However, these results do not suggest that dispersal from public surfaces to skin is the only, or even the major, driver of diurnal variation. Other regular patterns of activity, such as bathing, as well as physiological factors are very likely to play major roles.

Whilst there has been some previous work on the connection between the human circadian rhythm and the gut microbiome (reviewed in [24]), to our knowledge, there have been few previous reports on diurnal variation in the human skin microbiome, whether driven by physiological factors or by dispersal. Interestingly, one previous study examined human skin immediately after contact with public surfaces in the subway system in Hong Kong and found a similar pattern of diurnal variation between morning and evening samples, with the *Actinobacteria* as a major contributor to this variation as well as being the most abundant phylum overall [25]. This suggests that in addition to the effect of the daily rhythm of contact with public surfaces followed by ‘normalisation’ with the household microbiome that we propose above, there is also an effect of intra-day variation on public surfaces themselves, although as described above we found fewer significantly diurnal species on public surfaces than on skin or household surfaces.

Our results demonstrate yet another way in which forensic microbiota traces are not simply ‘microbial fingerprints’, but rather momentary snapshots of a dynamic network of microbial dispersal in which a person’s skin and a surface on which they leave a trace are only two nodes. In particular, they suggest that understanding the potential acquisition of microbiota from touched surfaces into the skin microbiome may be useful to develop effective matching methods. Despite the success of microbiota matching in this and previous similar studies [11–13], using relatively simple methods of comparing the community distances between reference and query samples, these studies have the advantage of identifying a match from a small and closed pool of references of which the correct match is known to be a member. In real-world forensic applications, the reference pool could potentially comprise the entire population of a city or country, with no certainty that the correct match is part of this pool. There have been some previous demonstrations of alternative approaches to microbial matching based on identifying marker taxa or sequences, including the metagenomic hitting set-based method described by Franzosa et al*.* [14], the hidSkinPlex panel of curated marker genes for forensic identification described by Schmedes et al. [26], and the use of minimum entropy decomposition (MED) to find individually identifying clusters of marker gene sequences described by Richardson et al. [27]. As we have shown in this and in our previous study [11], community-level matching will always have to contend with forces of microbial dispersal that degrade the similarity between a query and a reference sample. Approaches focused instead on identifying a targeted set of marker sequences or taxa that can then be traced through a dispersal event, or potentially even though multiple steps of a microbial dispersal network, may ultimately be more reliable for forensic matching than community-level methods.

## Conclusions

This study sought to examine the processes that affect microbiota matching accuracy on the timescale of hours to days. Our results reveal that these processes differ considerably between private and public surfaces, and are further complicated by diurnal variation and the time of day at which a sample was taken. There was good support for a model in which this diurnal variation is at least partially driven by dispersal from public surfaces to skin during daytime activity, followed by normalisation between skin and indoor surfaces at night, although other patterns of activity and physiological factors are very likely to play major roles. This study provides further evidence that the use of microbiome analysis in forensic identification is not a simple matter of comparing ‘fingerprints’, but rather depends on and must therefore account for dynamic systems of microbial dispersal.

## Methods

### Sample collection, DNA extraction and metagenome sequencing

Skin (left and right palm), indoor surface (bed headboard and entrance door knob) and public surface (outdoor park or campus handrail and subway exit handrail) swab samples were collected twice daily (morning collection between 8–9 a.m., evening collection between 5–8 p.m.) over a 10-day period in May 2018. Sampling locations included four residential units, each occupied by a single person i.e. not cohabiting with family or other occupants. The four people included in the study were not related and were instructed to follow their normal daily routines during the study period. For each residence, the handrails of an adjacent outdoor park or university campus and the nearest subway station exit were sampled as low- and high-traffic (respectively) public surfaces. These public surfaces were selected using a uniform process for each location, and were not intended to be surfaces known to be touched by the study participants nor were the participants instructed to touch these surfaces; rather, they were intended as representative of public surfaces in that area and therefore as reservoirs of the local population’s skin microbiome. MS Mini DNA/RNA Buccal Swabs (Isohelix, UK) were moistened with DNA/RNA Shield Reagent (Zymo Research, USA), and each surface (~5 cm^2^) was sampled with the moistened swab for 20 s using a standardised technique. Moistened swabs that were exposed to ambient air but not in contact with any surface were collected as negative control samples. Following sampling, swabs were immediately submerged in Shield Reagent and stored at –20 °C. Swabs were shipped to HudsonAlpha Genome Sequencing Center (AL, USA) for DNA extraction and Illumina paired-end 150 bp sequencing performed as described in Danko et al. [28]. Some samples were excluded from the study due to sequencing failure (Table S1).

### Taxonomic identification and decontamination

Adapters were removed from the raw sequences using AdapterRemoval (version 2.2.2, [29]). Quality filtering and trimming were performed using KneadData (https://github.com/biobakery/kneaddata, version 0.7.6) with default parameters, with human genome hg38 as the reference database to remove human sequences. This resulted in an average of 4,681,264 (62.1%) of reads retained from each sample. The microbial taxa in the negative controls were annotated using the metaphlan2.py script from MetaPhlAn2 [30], with the default database built from unique clade-specific marker genes. The genera *Bradyrhizobium* and *Staphylococcus* dominated all negative controls, with mean relative abundances of 50.7% and 13.8% respectively (Fig. S2). To remove the contaminant reads, reads from the 12 quality-filtered negative controls were co-assembled with metaWRAP (version 1.2.1, [31]) with megahit as the default assembly method, and a minimum contig length of 1000 bp. A bowtie2 (version 2.4.1, [32]) index was constructed from the assembled contigs using bowtie2-build with default settings, metagenome reads from skin and surface samples were aligned against this index using bowtie2 with default settings, and all reads that aligned were removed from further analysis. Unpaired reads were extracted from the paired-end fastq files using fastq-pair [33]. Following contaminant removal, an average of 2,001,606 reads per sample was retained. MetaPhlAn2 was used to compute the species abundances of the decontaminated skin and surface metagenomes as well as the negative control samples. A second decontamination step was then performed on these species-abundance profiles, using decontam (version 1.1.2, [34]) with the ‘prevalence’ method to identify species still present in the metagenomes that were likely contaminants based on their prevalence in the study samples compared to the negative control samples. Whilst 34 contaminant species were identified using this method and removed from the species-abundance profiles, these comprised on average only 3% of the relative abundance of each sample. Relative abundances were re-normalised to 100% following this step.

### Source analysis and microbiota matching

All downstream analysis was performed using R version 4.0.2 [35] with figures drawn using ggplot2 (version 3.3.2, [36]). SourceTracker (version 1.0.1, [20]) was used with default settings to identify likely sources for indoor and public surface communities, with skin and negative control communities as potential sources. Bray-Curtis dissimilarities and Canberra distances between samples, used for both the source analysis and for microbiota matching, were calculated with the vegdist() function from the R package vegan (version 2.5-7, [37]). Microbiota matching using SourceTracker was performed as previously described [11]. Briefly, ‘reference pools’ were constructed consisting of the set of all skin samples from each of the four people in the study at a given time point (i.e. a morning or evening sample from one day in the study). Time points without at least one skin sample for each person were excluded. SourceTracker was used for each surface sample ‘query’ to generate a set of source proportions from each reference pool, i.e. the estimated contribution of each sample in that reference pool to the query sample. The person with the highest estimated contribution to the query community was considered the match, and a match was considered accurate if that person was from the same location as the query (i.e. a sample from the residence occupied by that person, or from a public surface near their residence). To conform with the data formatting requirements of SourceTracker, which uses absolute species counts rather than relative abundances, species relative abundance percentages were multiplied by ten then rounded up to the nearest integer; this effectively removed species with relative abundances < 0.1%. Microbiota matching using Bray-Curtis dissimilarities and Canberra distances was performed using a modification of the method described by Watanabe et al. [17]. Briefly, a ‘reference pool’ was created for each time point as with the SourceTracker method, and for each ‘query’ surface sample, the reference sample with the smallest mean Bray-Curtis dissimilarity or Canberra distance from the query was considered to be the match, with accuracy defined as with the SourceTracker method. For all three methods, this approach allowed matching accuracy to be determined with sampling delays, as reference pools and query samples could be from any pairwise combination of time points.

### Time series decomposition and identification of diurnally varying species

PERMANOVA comparison of morning and evening communities was performed with the adonis2() function from the R package vegan [37], using the Bray-Curtis dissimilarity between communities. Time series decomposition of species abundances was performed in order to identify species that exhibited a significant diurnal pattern over the study period. Each time series was the relative abundance of a given species at a given site and location across the twenty time points (10 days) of the study period. Species that were not present for at least four time points were excluded from time series analysis. For sites and locations with missing samples for some time points (excluding at the beginning and end of the study period), abundances were interpolated using the na_seadec() function from the R package imputeTS version 3.1 [38]. Time series was performed with the built-in R function decompose(), which decomposes a time series into seasonal, trend and random (irregular) components using moving averages. The strength of the seasonal component *S*_*t*_ (*F*_*S*_ or ‘seasonality strength’) for each time series was calculated from the ratio between *S*_*t*_ and the random component *R*_*t*_ following Wang et al. [39]:

$$ {F}_S=\mathit{\max}\left(0,1-\frac{Var\left({R}_t\right)}{Var\left({S}_t+{R}_t\right)}\right) $$

To calculate the significance of *F*_*S*_, each time series was subjected to 999 random permutations of abundances, with each permuted time series then decomposed and *F*_*S*_ calculated. *p* was determined as the proportion of these permutations with *F*_*S*_ greater than the test *F*_*S*_ and significance defined as *p* < 0.05. This method for identifying significantly diurnal species was selected over pairwise tests of significance as it allows for decomposition of the trend component of the time series. For comparison, paired Mann-Whitney tests for a significant difference between morning and evening abundances were performed for all species at all sites. Using the same *p* < 0.05 threshold, of all species at a particular site and location identified as significantly diurnal using either method, the majority (95 species) were identified as significantly diurnal using both methods, whilst 65 species were identified using the time series method alone, and 34 species were identified using the Mann-Whitney method alone.

### Generation of dynamic Bayesian networks

Dynamic Bayesian networks (DBNs) were constructed to analyse the major routes of dispersal between the different types of sites in this study. Following the method of Lax et al. [13], species relative abundances within each sample were aggregated at the family level and log_*2*_-transformed. A total of 472 DBNs were generated, with each network representing one combination of location and taxonomic family. Each candidate network had six nodes, representing the six sites sampled in this study. Bayesian network inference with Java Objects (‘Banjo,’ https://users.cs.duke.edu/~amink/software/banjo/) was used to generate the networks with the following settings: no restrictions on network structure (i.e. no forbidden or mandatory edges); i5 discretisation policy; ‘Greedy’ searcher; ‘AllLocalMoves’ proposer; default evaluator and decider; minimum, maximum and mandatory Markov lags of 1 (i.e. all edges representing a single time-point increment); maximum parent count of 5 (the practical maximum in a network with 6 nodes); default stopping and simulated annealing criteria for a dynamic network.

## Supplementary Information

**Additional file 1: Supplementary Figure 1.** Effect of the time of day at which a query (surface) and/or reference pool (skin) were collected on microbiota matching accuracy.

**Additional file 2: Supplementary Figure 2.** taxonomic heatmap of the top 25 genera (y-axis) identified in 12 negative controls (x-axis).

**Additional file 3: Table S1.** summary of all samples collected for this study.

## Data Availability

Sequencing reads generated for this project have been deposited in NCBI under accession number PRJNA671748. Original R scripts are available in GitHub (https://github.com/wilkox/diurnal_variation).
