# Supplementary material for: Diurnal variation in the human skin microbiome affects accuracy of forensic microbiome matching
Source: Microbiome. 2021 Jun 5;9:129. doi: 10.1186/s40168-021-01082-1 (PMC8180031; doi:10.1186/s40168-021-01082-1)
Supplement: Supplementary file 2 — Additional file 1: Supplementary Figure 1. Effect of the time of day at which a query (surface) and/or reference pool (skin) were collected on microbiota matching accuracy. [file 40168_2021_1082_MOESM2_ESM.docx]

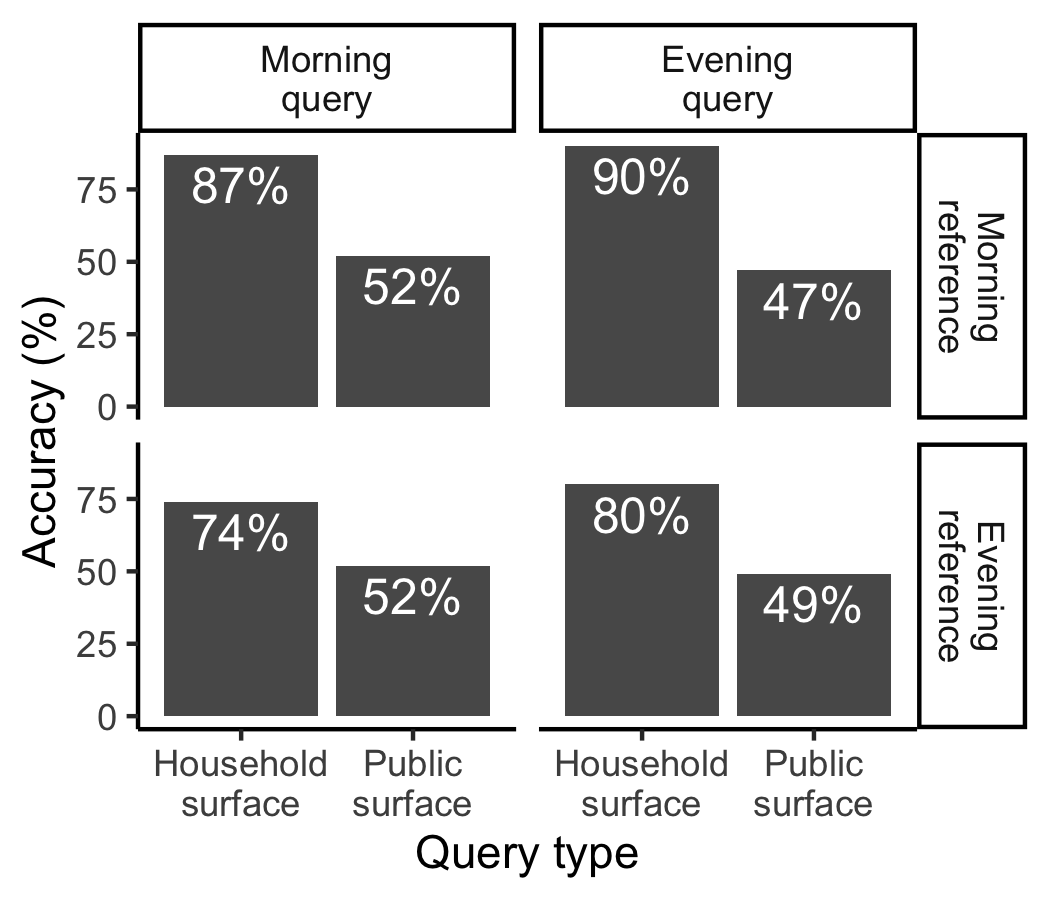


Supplementary Figure 1: Effect of the time of day at which a query (surface) and/or reference pool (skin) were collected on microbiota matching accuracy.
