# Supplementary material for: Diurnal variation in the human skin microbiome affects accuracy of forensic microbiome matching
Source: Microbiome. 2021 Jun 5;9:129. doi: 10.1186/s40168-021-01082-1 (PMC8180031; doi:10.1186/s40168-021-01082-1)
Supplement: Supplementary file 3 — Additional file 2: Supplementary Figure 2. taxonomic heatmap of the top 25 genera (y-axis) identified in 12 negative controls (x-axis). [file 40168_2021_1082_MOESM3_ESM.docx]

Supplementary Figure 2: taxonomic heatmap of the top 25 genera (y-axis) identified in 12 negative controls (x-axis).
