# Supplementary material for: Diurnal variation in the human skin microbiome affects accuracy of forensic microbiome matching
Source: Microbiome. 2021 Jun 5;9:129. doi: 10.1186/s40168-021-01082-1 (PMC8180031; doi:10.1186/s40168-021-01082-1)
Supplement: Supplementary file 4 — Additional file 3: Table S1. summary of all samples collected for this study. [file 40168_2021_1082_MOESM4_ESM.pdf]

Site	Surface material	Number of samples (by location)			
		Residence 1	Residence 2	Residence 3	Residence 4
Left palm	Skin	17	18	19	15
Right palm	Skin	16	16	20	13
Bed headboard	Mixed	18	17	20	12
Door knob	Mixed	17	16	20	11
Park/campus handrail	Metal	17	16	19	16
Subway exit handrail	Metal	16	16	20	13

Table S1: summary of all samples collected for this study.
